# MMP1 expression is activated by Slug and enhances multi-drug resistance (MDR) in breast cancer

**DOI:** 10.1371/journal.pone.0174487

**Published:** 2017-03-23

**Authors:** Ching-Ju Shen, Yu-Ling Kuo, Chien-Chung Chen, Ming-Jenn Chen, Ya-Min Cheng

**Affiliations:** 1 Graduate Institute of Medicine, College of Medicine, Kaohsiung Medical University, Kaohsiung, Taiwan; 2 Department of Gynecology and Obstetrics, Kaohsiung Medical University Hospital, Kaohsiung Medical University, Kaohsiung, Taiwan; 3 Department of Plastic and Reconstruction Surgery, E-Da Hospital, Kaohsiung, Taiwan; 4 Department of Surgery, Chi-Mei Medical Center, Tainan, Taiwan; 5 Department of Sports Management, College of Leisure and Recreation Management, Chia Nan University of Pharmacy and Science, Tainan, Taiwan; 6 Department of Obstetrics and Gynecology, Institute of Clinical Medicine, College of Medicine, National Cheng Kung University, Tainan, Taiwan; University of South Alabama Mitchell Cancer Institute, UNITED STATES

## Abstract

High matrix metalloproteinase 1 (MMP1) expression is associated with enhanced breast cancer growth and metastasis and also might predict poor prognosis. In this study, we further investigated the functional role of MMP1 and how it is upregulated in multi-drug resistant (MDR) breast cancer cells. By retrieving microarray data in GEO datasets and the survival data in the Kaplan Meier plotter, we observed that MMP1 is significantly upregulated in MCF-7/ADR cells compared to the parental MCF-7 cells, while high MMP1 expression is associated with worse overall survival (OS) and recurrence free survival (RFS) in breast cancer patients after systematic therapy. Functional studies showed that MMP1 overexpression significantly reduced the drug sensitivity in MCF-7 cells, while MMP1 knockdown substantially enhanced the sensitivity in MCF-7/ADR cells. By performing western blotting and immunofluorescent staining, we confirmed that MCF-7/ADR cells had enhanced mesenchymal properties than MCF-7 cells. In MCF-7 cells, enforced Slug expression resulted in significant MMP1 upregulation, while in MCF-7/ADR cells, Slug knockdown led to reduced MMP1 expression. By performing bioinformatic analysis, we observed that the promoter of MMP1 has three putative Slug binding sites. The following dual luciferase assay and ChIP-qPCR verified these three binding sites. Therefore, we infer that Slug enhances MMP1 transcription via directly binding to the promoter region in breast cancer cells, which is a previously unrecognized mechanism in the development of MDR.

## Introduction

Chemotherapy is the major therapeutic strategy for advanced breast cancer. However, nearly all initially responsive breast tumors will eventually develop the phenotype of multidrug resistance (MDR), which finally leads to therapeutic failure and cancer-related death [1–3]. The mechanisms of MDR are quite complex and far from been fully understood. Recent studies suggest that a series of dysregulated epigenetic modulations at transcriptional and post-transcriptional levels involve in induced adaptive responses of cancer cells to chemotherapeutic agents, such as epithelial mesenchymal transition (EMT) and cancer stem cells [4–7].

Matrix metalloproteinase 1 (MMP1), which is also known as interstitial collagenase and fibroblast collagenase is a member of the matrix metalloproteinases (MMPs) family [8]. Proteins in this family mainly participate in the breakdown of extracellular matrix both in normal physiological processes and disease processes [9]. High MMP1 expression might predict poor disease-free and overall survivals in patients with invasive breast carcinoma [10]. Functionally, MMP1 upregulation in breast cancer can enhance breast cancer growth and metastasis [11]. In adriamycin resistant MCF-7 cells (MCF-7/ADR), the expression of MMP1, MMP2 and MMP9 are significantly increased compared to the parental MCF-7 cells partly due to the upregulation of extracellular matrix metalloproteinase inducer [12]. However, the functional role of MMP1 in MDR breast cancer cells and whether other mechanisms are involved in its upregulation in the cells have not been fully understood.

One recent study suggest that the circulating breast tumor cells with EMT markers had significantly increased MMP1 expression [13]. In fact, the MDR breast cancer cells usually develop EMT phenotypes, with significant upregulation of several EMT-promoting transcription factors, such as Snail, Slug and Twist [4]. In this study, we found that MMP1 upregulation in breast cancer is associated with worse overall survival (OS) and recurrence free survival (RFS) in breast cancer patients after systematic therapy. MMP1 knockdown reduced the MDR of MCF-7/ADR cells. In addition, we demonstrated that Slug directly promotes MMP1 transcription, which is a previously unrecognized mechanism of MMP1 upregulation in MDR breast cancer cells.

## Materials and methods

### Bioinformatics analysis

Raw data of the gene microarray that assessed the gene transcriptional profile of doxorubicin-selected MCF-7/ADR cells and weakly tumorigenic parental MCF-7 cells was downloaded from NCBI GEO Datasets (accession No. GDS4084). The raw data was reanalyzed to identify the most upregulated and the most downregulated genes by using the Morpheus software (https://software.broadinstitute.org/morpheus/). The 100 most upregulated genes and 100 most downregulated genes in MCF-7/ADR cells were loaded into the Search Tool for the Retrieval of Interacting Genes (STRING) (http://string-db.org/) database for analysis of the protein-protein interaction network. To ensure the validity of the network, only experimentally validated interactions with high confidence score (≥ 0.70) were included.

The association between MMP1 expression and prognosis in all types of breast cancer patients and in ER+ breast cancer patients after systematic therapy was analyzed by using the Kaplan Meier plotter, which is an online database that provides assessment of the effect of 54,675 genes on survival using 10,293 cancer samples, including 22,277 genes in 5,143 breast cancer samples (http://kmplot.com/analysis/) [14, 15]. The association between MMP1 expression and metastatic relapse (MR) and any event (AE) free survival in breast cancer patients were further studied by using Breast Cancer Gene-Expression Miner Version 4.0 (bc-GenExMiner 4.0), a database of published annotated genomic data including 5609 breast cancer patients [16].

The possible binding site of Slug on the promoter region of MMP1 was predicted using the JASPAR Database (http://jaspar.genereg.net/).

### Cell culture and transfection

Human ER+ breast cancer cell line MCF-7 was purchased from the American Type Culture Collection (ATCC, Rockville, MD, USA). The adriamycin (ADR)-resistant MCF-7/ADR cells were generated by a conventional stepwise method that gradually increases the concentration of ADR over 8 months. The cancer cells were maintained in RPMI 1640 medium supplemented with 10% FBS, 2mM glutamine, 100 units of penicillin/ml and 100 μg of streptomycin/ml at 37°C and 5% CO_2_.

Human Slug (Snai2/Slug, NM_003068) cDNA ORF clone (cat. RG202363), Human MMP1 (NM_002421) cDNA ORF Clone (cat. RG202460) and the empty control pCMV6-AC-GFP vector (cat. PS100010) were purchased from OriGene (Rockville, MD, USA). MCF-7 cells were transiently transfected with either the Slug cDNA ORF clone, MMP1 cDNA ORF clone or pCMV6-AC-GFP vector using Lipofectamine 2000 (Invitrogen).

The ready-to-use MMP1 siRNA was purchased from Santa Cruz (sc-41552), while Slug siRNA (5’-GCUACCCAAUGGCCUCUCU-3’) [17] were chemically synthesized by Ribobio (Guangzhou, China). MCF-7/ADR cells were transfected with 40 nM MMP1 siRNA or Slug siRNA using Lipofectamine 2000 (Invitrogen).

### QRT-PCR analysis

Total RNA in the cell samples were extracted using the Trizol Reagent (Invitrogen) and then was reverse-transcribed using the iScript cDNA Synthesis kit (Bio-Rad, Hercules, CA, USA) according to the manufacturer's instructions. QRT-PCR was performed using gene specific primers (MMP1: F, 5’-CTTGCACTGAGAAAGAAGACAAAGG-3’, R 5’-ACACCCCAGAACAGCAGCA-3’; Slug: F, 5’-CACTATGCCGCGCTCTTC-3’, R 5'-GGTCGTAGGGCTGCTGGAA-3') and the SYBR^®^ Select Master Mix (Applied Biosystems) in an ABI 7900HT Fast Real-Time PCR System (Applied Biosystems). β-Actin was detected as the endogenous control.

### Drug sensitivity assay

24 h after indicating transfection, the cells were seeded in a 96-well plate and were further cultured overnight. Then, the cells with treated with varying concentrations of adriamycin, vincristine and paclitaxel for 48 h. After that, cell viability was measured using a conventional MTT (Sigma Aldrich) assay. Absorbance was recorded at 490 nm using a microplate reader. IC50 value was determined by creating dose-response curves.

### Flow cytometric analysis of caspase-9 activation

MCF-7 with or without MMP1 overexpression and MCF-/ADR cells with or without MMP1 knockdown were treated with adriamycin for 48 h. Then, the proportion of cells with activated caspase-9 was determined using the GaspGLOW^™^ Fluorescein Active Caspase-9 Staining Kit (Biovision, Mountain View, CA, USA) according to the manufacturer’s protocol. Fluorescence was excited with the 488-nm line of an argon laser on an EPICS XL flowcytometer (Beckman Coulter, Fullerton, CA, USA). At least 10,000 cells were analyzed for each sample. The results were shown as representative data of three repeated experiments.

### Western blotting analysis

Cell samples were lysed using a lysis buffer (Beyotime, Shanghai, China). Then, the protein concentration was quantified using a BCA protein assay kit (Beyotime). The samples containing 20 μg of proteins were subjected to separation in 10% SDS-PAGE and then transferred to polyvinylidene fluoride (PVDF) membranes. Primary antibodies used included anti-E-cadherin (ab15148, Abcam, Cambridge, UK), anti-N-cadherin (ab76057, Abcam), anti-Slug (ab27568, Abcam), anti-MMP1 (ab38929, Abcam) and anti-β-actin (Ab1801, Abcam). After that, the membranes were incubated with corresponding HRP conjugated secondary antibodies. The blot signals were visualized using the ECL Western blotting substrate (Promega, Madison, WI, USA). The relative expression of the proteins were quantified using ImageJ software.

### Fluorescence microscopy

MCF-7 cells and MCF-7/ADR cells were cultured on coverslips. Then, the cells were fixed, permeabilized in 0.1% Triton X-100 and blocked in 1% BSA. The coverslips were incubated with anti-N-cadherin (ab76057, Abcam) or anti-Slug (ab27568, Abcam) at 4°C overnight. Alexa Fluor^®^ 488 Conjugate Secondary Anti-Rabbit (#4412, Cell Signaling) was used to detect N-cadherin, while Alexa Fluor^®^ 555 Conjugate Secondary Anti-Rabbit (#4413, Cell Signaling) was used to detect Slug. The coverslips were incubated with the secondary antibodies for 1 h at room temperature in the dark. Nuclei were stained using Prolong^®^ Gold Antifade Reagent with DAPI (#8961, Cell Signaling). Immunofluorescent images were obtained using an Olympus IX81 inverse microscope (Tokyo, Japan). Each experiment was performed in triplicate.

### Dual luciferase assay

The MMP1 promoter plasmid was obtained from GeneCopoeia (cat. HPRM20638). The MMP1 promoter is a 1326 bp region that contains 1255 bp upstream and 70 bp downstream of the transcriptional start site (TSS) site. The sequence of the promoter was given in S1 Table. The predicted three Slug binding sites located on -741 to -733, -424 to -416 and -406 to -398 upstream the TSS site. Five truncated promoter sequences, including -741 to +71, -700 to +71, -500 to +71, -410 to +71 and -300 to +71 were PCR amplified from the MMP1 promoter plasmid. The promoter fragments were then cloned into the *XhoI*-*Hind III* site of the pGL3-basic luciferase reporter vector respectively. MCF-7 cells were seeded into 12-well plates at a density of 1×10^5^ cells/well and were co-transfected the with 1.5 μg luciferase construct plasmid or the empty reporter vector DNA and 0.05 μg phRL-TK by using Superfectin (Qiagen, Valencia, CA, USA). 24 h after transfection, cells were lysed and the luciferase activity was assessed using the dual-luciferase reporter assay system (Promega) with a luminometer (Promega). The luciferase activity was normalized to the activity of renilla luciferase.

### Chromatin Immunoprecipitation assay (ChIP)

ChIP assay was performed by using the Upstate-ChIP Assay Kit (Lake Placid, NY, USA) following the manufacturer's instructions. In brief, DNA from MCF-7 and MCF-7/ADR cells was submitted to immunoprecipitation with 4 μg of normal goat IgG or anti-Slug (sc-10436, Santa Cruz Biotechnology). Then, ChIP-enriched DNA was analyzed by qRT-PCR using the ABI PRISM 7900HT sequence detection system and SYBR green master mix. Primers used were: GAPDH, 5'-agcgcaggcctcaagacctt-'3 (forward) and 5'-aagaagatgcggctgactgt-3' (reverse), and MMP1 promoter, 5’-ttttaatgggcaggagatgc-3’ and 5'-ggatgatgaaaaggctggaa-3' (reverse).

### Statistical analysis

Data were presented in the form of means ± standard deviation (SD). Data were analyzed for statistical significance by two-tailed Student’s t test or ANOVA with Student-Newman-Keuls test as a post hoc test. p value of <0.05 was considered statistically significant.

## Results

### MMP1 is upregulated in MCF-7/ADR cells compared to the parental MCF-7 cells

**Fig 1:**
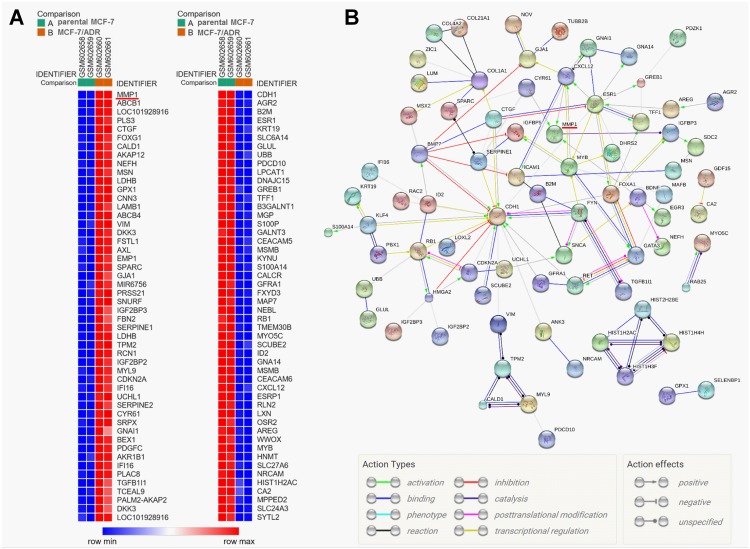
MMP1 is upregulated in MCF-7/ADR cells compared to the parental MCF-7 cells. **A**. Heat map of the top 50 upregulated genes (left) and 50 downregulated genes (right) in MCF-7/ADR cells compared to the parental MCF-7 cells. Red: up-regulation; Blue: down-regulation. Raw microarray data was obtained from GEO dataset (accession: GDS4084). **B**. The protein-protein interaction (PPI) network of the 100 most upregulated and the 100 most downregulated genes analyzed by using the (STRING) (http://string-db.org/) database. Only experimentally validated interactions with a high confidence score ≥ 0.70 were included.

One previous microarray data (GDS4084) compared the dysregulated genes in MCF-7/ADR cells compared to the parental MCF-7 cells. The raw data of this microarray was downloaded for reanalysis of the dysregulated genes. The heat maps in Fig 1A showed the 50 most upregulated (left) and the 50 most downregulated (right) genes in MCF-7/ADR cells compared to the parental MCF-7 cells. Among the dysregulated genes, MMP1 is among of the most upregulated genes, while CDH1 is among of the most downregulated genes (Fig 1A, left). By performing protein-protein interaction network analysis, we observed that CDH1 is a hub gene in the network (Fig 1B). In fact, multiple previous studies reported that breast cancer cells with enhanced MDR had decreased epithelial properties and increased mesenchymal properties [2, 5, 18]. However, the functional role of MMP1 in MDR of breast cancer cells is not well characterized. In addition, how dysregulated MMP1 is related to the EMT of MDR breast cancer cells have not been fully revealed.

### High MMP1 expression is associated with worse survival outcomes in breast cancer patients after systematic therapy

**Fig 2:**
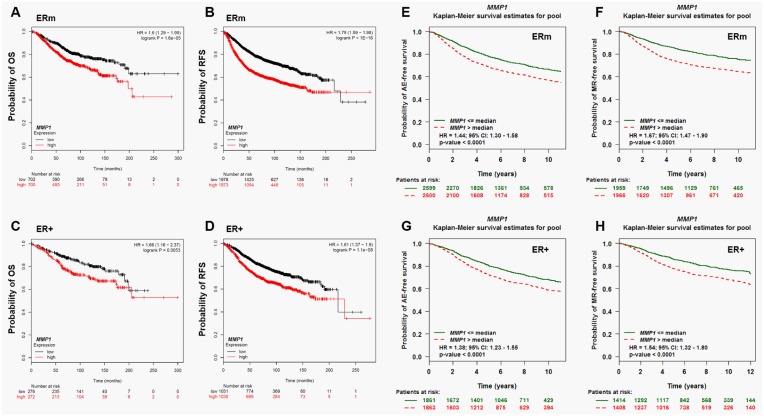
High MMP1 expression is associated with worse survival outcomes in breast cancer patients after systematic therapy. **A-D**. The OS curves (A and C) and RFS curves (B and D) of all breast cancer patients (A and B) and in ER+ breast cancer patients (C and D) with high or low MMP1 expression. Data was retrieved from the Kaplan Meier plotter (http://kmplot.com/analysis/). **E-G**. Kaplan-Meier survival analysis showing the relationship between MMP1 expression and AE-free survival (E and G) and MR-free survival (F and H) in breast cancer patients using bc-GenExMiner 4.0. ERm: mixed ER status.

Then, we examined whether MMP1 expression is related to prognosis in breast cancer patients after systematic therapy (including endocrine therapy and chemotherapy) by retrieving the data in the Kaplan Meier plotter. The results showed that high MMP1 expression is associated with significantly worse OS and RFS no matter in all breast cancer patients (Fig 2A and 2B) or only in ER+ breast cancer patients (Fig 2C and 2D). To further verify the findings, we examined the association between MMP1 expression and AE-free survival and MR-free survival among breast cancer patients by using bc-GenExMiner 4.0. The results also suggested that high MMP1 expression is associated with significantly worse AE-free survival and MR-free survival no matter in all breast cancer patients (Fig 2E and 2F) or only in ER+ breast cancer patients (Fig 2G and 2H).

### MMP1 upregulation results in enhanced MDR in MCF-7 cells

**Fig 3:**
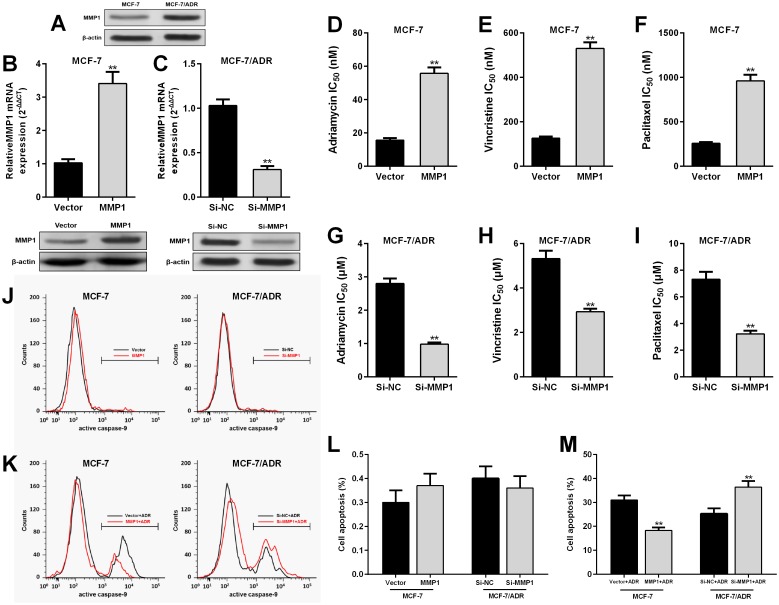
MMP1 upregulation results in enhanced MDR in MCF-7 cells. **A**. Western blotting of MMP1 expression in MCF-7/ADR and MCF-7 cells. **B-C**. QRT-PCR analysis of MMP1 mRNA (up) and western blotting (down) of MMP1 protein expression in MCF-7 cells 24 h after transfection of MMP1 expression vector or the empty control (B) and in MCF-7/ADR cells 24 h after transfection of MMP1 siRNA or the empty control (C). **D-I**. Adriamycin, vincristine and paclitaxel IC50 in MCF-7 cells with or without MMP1 expression (D-F) and in MCF-7/ADR cells with or without MMP1 knockdown (G-I). **J-K**. Representative flow cytometric images of cells with active caspase-9 in MCF-7 cells with or without MMP1 overexpression and in MCF-/ADR cells with or without MMP1 knockdown (J) and in the cells with combined adriamycin treatment (20 nM for MCF-7 cells and 1 μM for MCF-7/ADR cells) for 48 h (K). **L-M**. The proportion of cells with active caspase-9 showed in J (L) and K (M). ** p<0.01.

Considering the possible involvement of MMP1 in therapy response among breast cancer patients, we decided to further studies the underlying mechanisms. Western blot assay confirmed that MCF-7/ADR cells had significantly higher MMP1 expression than the parental MCF-7 cells (Fig 3A). MCF-7 cells were transfected with MMP1 expression vector for overexpression (Fig 3B), while MCF-7/ADR cells were transfected with MMP1 siRNA for knockdown (Fig 3C). The IC50 values of adriamycin, vincristine and paclitaxel in MCF-7 cells all drastically increased after MMP1 overexpression (Fig 3D–3F), but were significantly decreased in MCF-7/ADR cells after MMP1 knockdown (Fig 3G–3I). To further verify the effect of MMP1 on drug response, MCF-7 cells and MCF-7/ADR cells were subjected to flow cytometric analysis of active caspase-9 expression with or without adriamycin treatment. The results showed that MMP1 overexpression or knockdown had no effect on caspase-9 activation (Fig 3J and 3L). However, MMP1 overexpression significantly reduced adriamycin induced cell apoptosis in MCF-7 cells, while MMP1 knockdown substantially enhanced adriamycin induced cell apoptosis in MCF-7/ADR cells (Fig 3K and 3M).

### Slug modulates MMP1 expression in MCF-7/ADR cells

**Fig 4:**
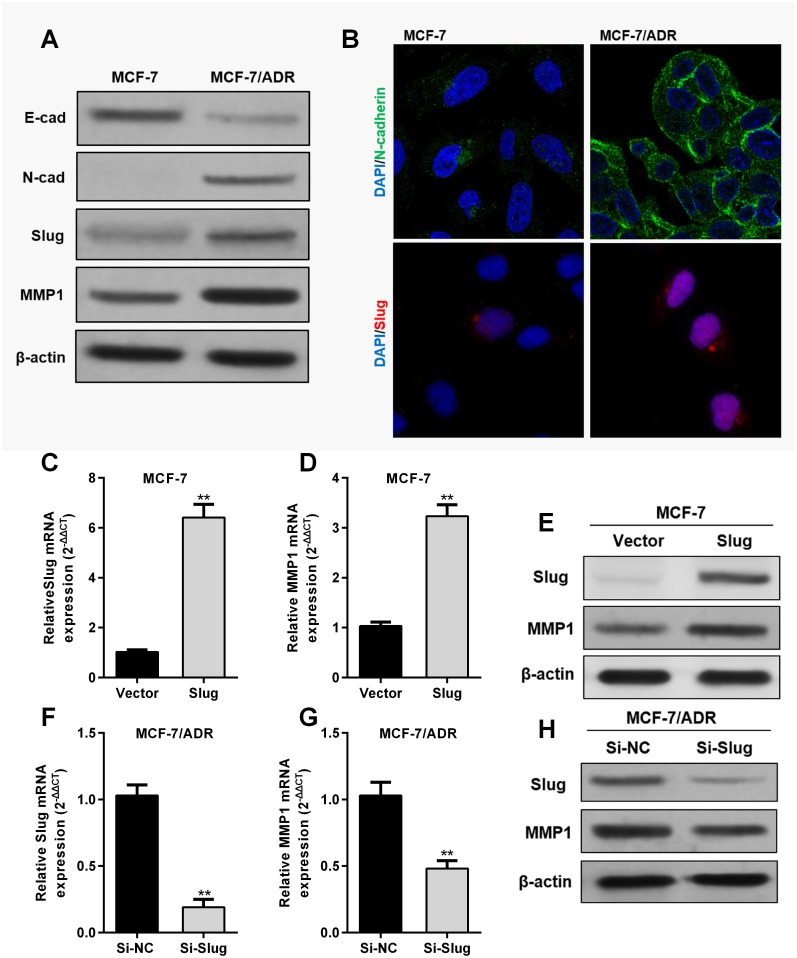
Slug modulates MMP1 expression in MCF-7/ADR cells. **A**. Western blotting of E-cadherin, N-cadherin, Slug and MMP1 expression in MCF-7 cells and in MCF-7/ADR cells. **B**. Immunofluorescent staining of N-cadherin and Slug in MCF-7 and in MCF-7/ADR cells. Green: N-cadherin; Green: Slug; Blue: DAPI. **C-H**. QRT-PCR analysis of Slug (C and F) and MMP1 (D and G) mRNA expression and western blotting of Slug and MMP1 protein expression (E and H) in MCF-7 cells with or without Slug overexpression (C-E) and in MCF-7/ADR cells with or without Slug knockdown (F-H). ** p<0.01.

By performing western blotting, we confirmed that MCF-7/ADR cells had significantly decreased E-cadherin (Fig 4A), but with increased N-cadherin compared to MCF-7 cells (Fig 4A and 4B). In addition, we also found that Slug, a transcription factor that facilitates EMT, is upregulated in MCF-7/ADR cells than in MCF-7 cells (Fig 4A and 4B). Interestingly, in MCF-7 cells, enforced Slug expression resulted in significant MMP1 upregulation (Fig 4C–4E), while in MCF-7/ADR cells, Slug knockdown led to reduced MMP1 expression (Fig 4F–4H).

### Slug upregulation leads to aberrant MMP1 expression via directly binding to the promoter of MMP1

**Fig 5:**
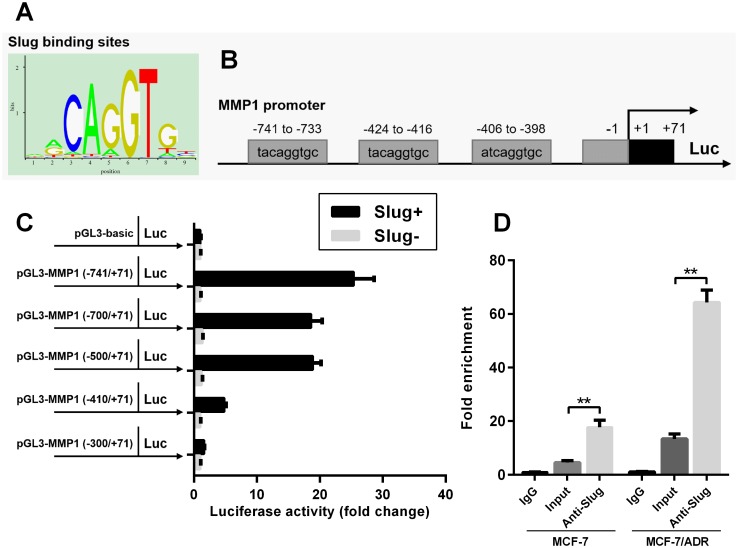
Slug upregulation leads to aberrant MMP1 expression via directly binding to the promoter of MMP1. **A-B**. Slug binding positions (A) and the predicted Slug binding sites in the promoter region of MMP1 (B). **C**. The luciferase reporter constructs of the truncated MMP1 promoters were introduced into HEK-293 cells transfected with Slug cDNA ORF clone or the empty control. Luciferase activity was measured 48 h post-transfection. **D**. Fold-enrichment of Slug binding at the MMP1 promoter relative to background in MCF-7 and MCF-7/ADR cells were measured by qRT-PCR. Upon normalization to GAPDH, results were expressed as n-fold compared to IgG. Data are expressed as mean ± SD from three independent experiments. ** p<0.01.

Bioinformatic analysis showed that there are three possible Slug binding sites located between -741 to -733, -424 to -416 and -406 to -398 bp on the MMP1 promoter (Fig 5A and 5B). Therefore, we hypothesized that Slug can act on the MMP-1 promoter and enhance its expression. To verify the hypothesis, we generated luciferase reporter constructs carrying the truncated MMP-1 promoters. Truncation of any of the three predicted Slug binding site resulted in a significant decrease in transcription, suggesting that the three binding sites are all important for Slug mediated MMP1 upregulation (Fig 5C) However, one truncated promoter (-300 to +71) did not display any transcriptional activity (Fig 5C). To further verify the direct binding of Slug to MMP1 promoter, ChIP with following qRT-PCR were performed. The results confirmed that Slug can effectively bind to the MMP1 promoter in both MCF-7 and MCF-/ADR cells (Fig 5D.)

## Discussion

MMPs are calcium-dependent zinc-containing endopeptidases that involve in cell behaviors such as cell proliferation, migration, differentiation, angiogenesis, apoptosis, and host defense [19, 20]. MMP1, together with MMP8 and MMP13 are collagenases are capable of degrading triple-helical fibrillar collagens into distinctive 3/4 and 1/4 fragments [21, 22]. In this study, by reviewing one available microarray data, we observed that MMP1 is significantly upregulated in MCF-7/ADR cells compared to the parental MCF-7 cells.

Previous studies mainly focused on the role of MMP1 in cancer cell growth, invasion and metastasis. In breast cancer, some recent studies reported that MMP1 upregulation is associated with more aggressive phenotype of breast cancer. For example, MMP1 upregulation in breast cancer spheroids can lead to paracrine PAR1 activation and disintegration in the lymph endothelial barrier in vitro [23]. MMP1 elevation can also promote the local growth and the formation of brain metastases of breast cancer cells [11]. Knockdown of MMP1 inhibited the growth and invasive ability of breast cancer cells in vitro, and also attenuated brain metastasis and lung metastasis in vivo [11]. In addition, some studies also found that high MMP1 expression might predict poor disease-free and overall survivals in patients with invasive breast cancer [10, 24]. In this study, by reviewing the data in the Kaplan Meier plotter and bc-GenExMiner 4.0, we observed that high MMP1 expression is associated with significantly worse survival outcomes no matter in all breast cancer patients or only in ER+ breast cancer patients. However, limited studies investigated the involvement of MMP1 in drug responses of the cancer cells. Therefore, we further investigated the functional role of MMP1 in drug responses of the cancer cells. The results showed that MMP1 overexpression significantly reduced the drug sensitivity in MCF-7 cells, while MMP1 knockdown substantially enhanced the sensitivity in MCF-7/ADR cells.

The mechanism of MMP1 upregulation in breast cancer is quite complex. Previous studies suggest that Cdc25A can increase Foxo1 stability, while Foxo1 can enhance transcription of MMP1, leading to enhanced breast cancer cell metastasis [25]. NF-kappaB can also significantly stimulate MMP1 upregulation in breast cancer cells [23]. In addition, upregulation of extracellular matrix metalloproteinase inducer may also result in increased expression of MMP1, MMP2 and MMP9 in MCF-7/ADR cells compared to the parental MCF-7 cells [12]. In the current study, we further investigated whether other mechanisms contribute to MMP1 upregulation in MCF-7/ADR cells. The MDR cancer cells usually develop EMT phenotypes, with significantly upregulation of several EMT-promoting transcription factors, such as Snail, Slug and Twist [4]. In addition, one recent study observed that the circulating breast tumor cells with EMT markers had significantly increased MMP1 expression [13]. These findings suggest that MMP1 expression might be related to EMT of the cancer cells. In this study, we observed that MCF-7/ADR cells had enhance mesenchymal properties. In addition, enforced Slug expression substantially increased MMP1 level in MCF-7 cells, while knockdown of Slug significantly decreased MMP1 level in MCF-7/ADR cells. By performing bioinformatic analysis, we observed that the promoter of MMP1 has three putative Slug binding sites. The following dual luciferase assay verified these three binding sites all had transcription activity. Based on these findings, we infer that slug can activate MMP1 transcription via directly binding to the promoter region.

In fact, the role of Slug in drug resistance in breast cancer has been reported in previous studies. Slug can induce elevation of cyclin D1 in breast cancer cells [26], which is an important mediator of both tamoxifen resistance and MDR [27–29]. Knockdown of Cyclin D1 could abolish the MDR phenotypes of breast cancer cells [29]. SiRNA targeting Slug can also enhance chemosensitivity of MDA-MB-231 cells via inhibiting the PI3K/Akt/GSK3beta signaling pathway [30]. However, as a transcriptional factor, Slug may regulate multiple molecular pathways. In combination with our findings, we infer that MMP1 is one of the downstream effectors of Slug in modulating chemosensitivity of breast cancer.

## Conclusion

MCF-7/ADR cells had significantly upregulated MMP1 expression than the parental MCF-7 cells. High MMP1 expression is associated with worse survival outcomes in breast cancer patients after systematic therapy. MMP1 knockdown reduced the MDR of MCF-7/ADR cells. Slug directly promotes MMP1 transcription, which is a previously unrecognized mechanism of MMP1 upregulation in MDR breast cancer cells.

## Supporting information

S1 Table The sequence of MMP1 promoter. (DOCX)
