# Supplementary material for: MMP1 expression is activated by Slug and enhances multi-drug resistance (MDR) in breast cancer
Source: PLoS One. 2017 Mar 23;12(3):e0174487. doi: 10.1371/journal.pone.0174487 (PMC5363985; doi:10.1371/journal.pone.0174487)
Supplement: S1 Table — (DOCX) [file pone.0174487.s001.docx]

>HPRM20638 NM_002421; name=MMP1;Entrez_ID=4312;Genome=hg18;chr11-:102175359-102174034;TSS=102174104;Upstream=1255,Downstream=70;Length=1326;

ggaatacacatctttcattacagagccatgtatttattttaatgggcaggagatgctaaa

taagatcttttgaatggaggaatgcataaatatatgaatgaatgcatacatgaaagaata

aataaatgctgcctagcaccaaggagcgaagatagactcatatcaagggaaacaagtatg

attaaaaataagaccccagagtcacgctcagtctctttccagccttttcatcatccggta

cattcagacaagtttcagggaaggatcctatttgtcccatgataatgatgggcaaggggt

ggggagttatctcatactccgcctgtggatgaggggtcttctcaggtaaggctcttaaat

cctaggcctgagtaaattttttcaaattttattttagacagggtccctctctgttgccta

ggctggagtgcagcggcacaatcacagctcaatgcagcctcaacctcccaggcccaagtg

atcctcccacctcagcctcttcagtgactaggactacaggtgcatgactccatgcttggc

taactttaaaaaatgtttgtttgtttgtttgttttttacagagatggggtctcaccatgt

tgcccaggctgatcttgaactcctgggctcaagtgattcccctgcctcggcctcctgaaa

ttctgggattataggcttgagccaccatgcctggctctgagtaaagattaagggaagcca

tggtgctatcgcaatagggtaccaggcagcttaacaaaggcagaagggaacctcagagaa

ccccgaagagccaccgtaaagtgagtgctgggggagctgaacttcagtcagtacaggtgc

cgaacagccatcaggtgcgcagtgttagtaattccaccctctgccctgggagcaaggtgt

gtggagaaacctgtagcactttatgaccatcagaaccagtctttttcaaaaagaccatgg

agtactctttgacctgtgtatataacaagaacctttctcaaataggaaagaaatgaattg

gagaaaaccactgtttacatggcagagtgtgtctccttcgcacacatcttgtttgaagtt

aatcatgacattgcaacaccaagtgattccaaataatctgctaggagtcaccatttctaa

tgattgcctagtctattcatagctaatcaagaggatgttataaagcatgagtcagacagc

ctctggctttctggaagggcaaggactctatatatacagagggagcttcctagctgggat

attggagcagcaagaggctgggaagccatcacttaccttgcactgagaaagaagacaaag

gccagt
